# Cadaveric Evaluation of Different Approaches for Quadratus Lumborum Blocks

**DOI:** 10.1155/2018/2368930

**Published:** 2018-06-11

**Authors:** Hun-Mu Yang, Sang Jun Park, Kyung Bong Yoon, Kyoungun Park, Shin Hyung Kim

**Affiliations:** ^1^Department of Anatomy, Yonsei University College of Medicine, Seoul, Republic of Korea; ^2^Department of Anesthesiology and Pain Medicine, Anesthesia and Pain Research Institute, Yonsei University College of Medicine, Seoul, Republic of Korea

## Abstract

**Background:**

A quadratus lumborum (QL) block is an abdominal truncal block technique that primarily provides analgesia and anaesthesia to the abdominal wall. This cadaveric study was undertaken to compare the dye spread between different needle approaches for ultrasound-guided QL blocks in soft-embalmed cadavers.

**Methods:**

After randomization, an experienced anesthesiologist performed two lateral, three posterior, and five alternative QL blocks on the left or right sides of five cadavers. The target injection point for the alternative approach was the lumbar interfascial triangle, same as that of conventional posterior QL block, with a different needle trajectory. For each block, 20 ml of dye solution was injected. The lumbar region and abdominal flank were dissected.

**Results:**

Ten blocks were successfully performed. Regardless of the approach used, the middle thoracolumbar fascia was deeply stained in all blocks, but the anterior layer was less stained. The alternative approach was more associated with spread of injectate to the transversus abdominis and transversalis fascia plane. Despite accurate needle placement, all lateral QL blocks were associated with a certain amount of intramuscular or subcutaneous infiltration. Two posterior QL blocks showed a deeply stained posterior thoracolumbar fascia, and one of them was associated with obvious subcutaneous staining. The subcostal, iliohypogastric, and ilioinguinal nerves were mostly involved, but the thoracic paravertebral space and lumbar plexus were not affected in all blocks.

**Conclusions:**

The alternative approach for QL blocks was able to achieve a comparable extent when compared to the conventional approach.

## 1. Introduction

A quadratus lumborum (QL) block is an abdominal truncal block technique that primarily provides analgesia and anaesthesia to the abdominal wall [1, 2]. Although this technique has similarities to the posterior transversus abdominis plane (TAP) block, the extent of its effects has been suggested to be greater because the target injection point is more dorsal and the potential cephalad spread of local anaesthetics could reach the thoracic paravertebral space [3, 4].

Currently, at least three different approaches for QL blocks have been introduced in clinical practice [1, 5]. With an anteroposterior needle trajectory, the target injection point of a lateral QL block is the anterolateral margin of the QL, and that of a posterior QL block is the lumbar interfascial triangle (LIFT) on the posterior surface of the QL [1, 5]. Meanwhile, an anterior QL block is performed in the plane between the QL and the psoas muscle with the posteroanterior needle trajectory [5, 6].

A recent cadaver study reported that deep back muscle injection or spread of injectate posteriorly to the subcutaneous tissue can occur in lateral and posterior QL blocks [7]. We hypothesized that different needle trajectories could have spread less to the subcutaneous layer or intramuscular infiltration. Thus, we designed a different approach with a posteroanterior needle trajectory for the posterior QL block towards the LIFT along the posterolateral margin of the erector spinae (ES).

This study was undertaken to compare the spread of injectate between the conventional lateral and posterior QL block, and the posterior QL block with a different needle trajectory in soft-embalmed cadavers. Ultimately, we sought to evaluate whether the posterior QL block with a different needle trajectory could achieve an extent in the spread of injectate similar to that seen with the conventional approach for QL blocks and could reduce the spread to the subcutaneous layer or intramuscular infiltration.

## 2. Materials and Methods

This cadaveric anatomical study was approved by the institutional review board of Severance Hospital, Yonsei University Health System (ref. no. 4-2017-0553). All cadavers utilized in the present study were legally donated to the Surgical Anatomy Education Center at Yonsei University College of Medicine (YSAEC). Five soft-embalmed cadavers were acquired; the soft-embalmed cadavers were fixed by the Thiel method (17% ethylene glycol, 11% ammonium nitrate, 3% chlorate kersol, and 2% formaldehyde) [8]. Ten QL blocks were conducted on 5 cadavers independently chosen by the scientific officer of the YSAEC. Cadavers were randomized either to lateral or posterior QL blocks or to the posterior QL block with a different needle trajectory.

### 2.1. Ultrasound-Guided QL Block Procedure

All cadavers were placed in the lateral position, and blocks were performed by a single-experienced anesthesiologist with specialty in regional anesthesia and pain medicine. An M-Turbo ultrasound unit (SonoSite Inc, Bothell, WA, USA) with a convex probe (2–5 MHz) and an 80 mm, 22-gauge needle were used for every cadaver. The needle was attached to an extension tube and connected to a syringe containing a 20 ml mixture of 17.75 ml distilled water, 2 ml latex solution, and 0.25 ml blue-green coloured ink. This composition of injectate was chosen based on the results of a previous cadaver study [7].

For all blocks, the ultrasound probe was placed transversely at the L3-L4 level above the iliac crest. In this position, the ES (paraspinal muscles: multifidus, longissimus, and iliocostalis), which is contained within the paraspinal retinacular sheath, was identified, and the probe was then moved laterally to identify the QL, located below the ES and the latissimus dorsi (LD) muscle and superficial to the psoas muscle. At this point, the LIFT between the QL and the ES and the LD within the middle layer of the TLF were also identified [8]. Then, the probe was moved anterolaterally in order to visualize the anterolateral margin of the QL and the aponeurosis of the two abdominal wall muscles (IO: internal oblique muscle; TA: transversus abdominis muscle).

**Figure 1:**
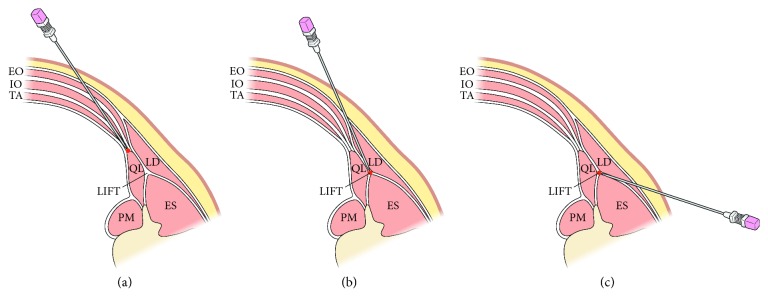
Illustration demonstrating the needle trajectory and tip location of the three different approaches to the ultrasound-guided QL block. With an anteroposterior needle trajectory, the target injection point of a lateral QL block (a) is the anterolateral margin of the QL, and that of posterior QL block (b) is the LIFT on the posterior surface of the QL. For a posterior QL block with posteroanterior needle trajectory (an alternative approach) (c), the needle is advanced towards the LIFT on the posterior surface of the QL (ES, erector spinae; LD, latissimus dorsi; LIFT, lumbar interfascial triangle; QL, quadratus lumborum; PM, psoas major; EO, external oblique muscle; IO, internal oblique muscle; TA, transversus abdominis muscle).

**Figure 2:**
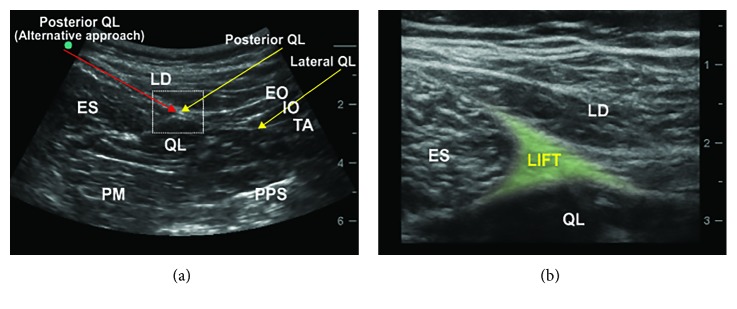
(a) Ultrasound image demonstrating the needle trajectory and tip location of the three different approaches to the ultrasound-guided QL block. (b) Ultrasound image of the lumbar interfascial triangle (LIFT) (ES, erector spinae; LD, latissimus dorsi; QL, quadratus lumborum; EO, external oblique muscle; IO, internal oblique muscle; TA, transversus abdominis muscle; PM, psoas major; PPS, posterior pararenal space).

Each approach for QL blocks is illustrated in Figure 1. For the lateral QL block, the needle was advanced to the anterolateral surface of the QL, immediately lateral to the tapered end of the TA muscle, after passing the abdominal wall muscles with an anteroposterior needle trajectory (lateral-to-medial direction) using the in-plane technique under ultrasound guidance (Figure 2). The target point of the posterior QL block was the LIFT, which is more superficially located on the posterolateral aspect of the QL. In a similar fashion to the lateral QL block, the needle was inserted towards the LIFT with the in-plane technique under ultrasound guidance (Figure 2).

For the posterior QL block with a different needle trajectory (an alternative approach) used in this study, the needle was first inserted between the ES and the LD. The needle was then advanced towards the posterior surface of the QL, locating the LIFT with a posteroanterior needle trajectory (medial-to-lateral direction). The final needle tip placement for the alternative approach in this study was the same as that of the conventional posterior QL block (Figure 2).

After injection of 1 to 2 ml of normal saline to confirm the correct localization of the needle tip in all blocks, a total of 20 ml of injectate mixture were injected for two minutes on each side of the cadavers. At completion of the injection, the needle was finally withdrawn after flushing out the dye. Sonographic images were recorded digitally in all cadavers.

### 2.2. Cadaveric Anatomic Dissection

On the next day, the cadavers were dissected in layers, from superficial to deep, with subsequent verification of the extent of dye spread. The skin and subcutaneous tissue on the abdominal flank, spreading horizontally from the midline to the midaxillary line and vertically from the 10th thoracic vertebra to the sacrum, were removed to reveal the LD, external oblique muscle (EO), and the posterior layer of the TLF. A flap of the LD and ES was raised serially, and the QL and two lowest ribs with their intercostal muscles were meticulously exposed with the middle layer of the TLF. Laterally, the IO was stripped off, and then, the TAP was revealed on the TA muscle. After removal of the TA muscle, the anterior layer of the TLF and transversalis fascia (TF) lining on the inside of the QL and TA muscle was exposed on the posterior pararenal space. The extent of dye spread was determined within the subcutaneous layer, TLF, TAP, TF, posterior paraspinal space, and space anterior to the psoas muscle. Superiorly, the intercostal spaces, with layers between the intercostal muscles, endothoracic membrane, and parietal pleura were examined after removal of the lower intercostal muscles. Inferiorly, the iliac fossa, to which the middle layer of the TLF is attached, was also examined. Additionally, the spinal nerves, comprising T11 (the 11th intercostal nerve), T12 (the subcostal nerve), and L1 (iliohypogastric and ilioinguinal nerves) were localized, and the staining of these nerves was evaluated. To determine the uppermost and foremost position of the dye spread, the thoracic paravertebral space and arcuate ligaments of the diaphragm were examined. To simplify and standardize the results, the degree of staining of these anatomical structures was agreed upon by consensus between an anatomist (HMY) and an anesthesiologist (SHK) and defined as either deeply stained, faintly stained, or unstained. Stained areas were recorded in brief sketches, notes, and photographs, documenting their relationship to fascial planes.

### 2.3. Statistical Analysis

Considering the nature of this cadaveric study, the extent of dye spread could not be precisely quantified in all anatomical structures. Thus, relatively well-distinguished spread patterns are presented as proportions (%) with 95% confidence intervals (CIs). The numbers of stained nerves among subcostal, iliohypogastric, and ilioinguinal nerves were compared between conventional and alternative approaches using the Mann–Whitney *U* test. Data were analysed using the Statistical Package for the Social Sciences 23.0 (SPSS Inc, Chicago, IL, USA). A *P* value less than 0.05 was considered statistically significant.

## 3. Results

In total, five soft-embalmed cadavers were included in this study. The subjects included three males and two females, with an average age of 76 years. The key sonographic landmarks were readily identified in all posterior to lateral abdominal walls of all cadavers. The QL blocks were successfully performed on each side of all five cadavers (10 sides) including 2 lateral QL blocks, 3 posterior QL blocks, and 5 alternative QL blocks (the posterior QL block with a different needle trajectory). Among them, the LIFT with a typical triangle shape was not found in four sides; however, the intersecting point between the ES, the LD, and the QL was clearly demarcated in all 10 sides. A summary of block characteristics is given in Table 1.

**Figure 3:**
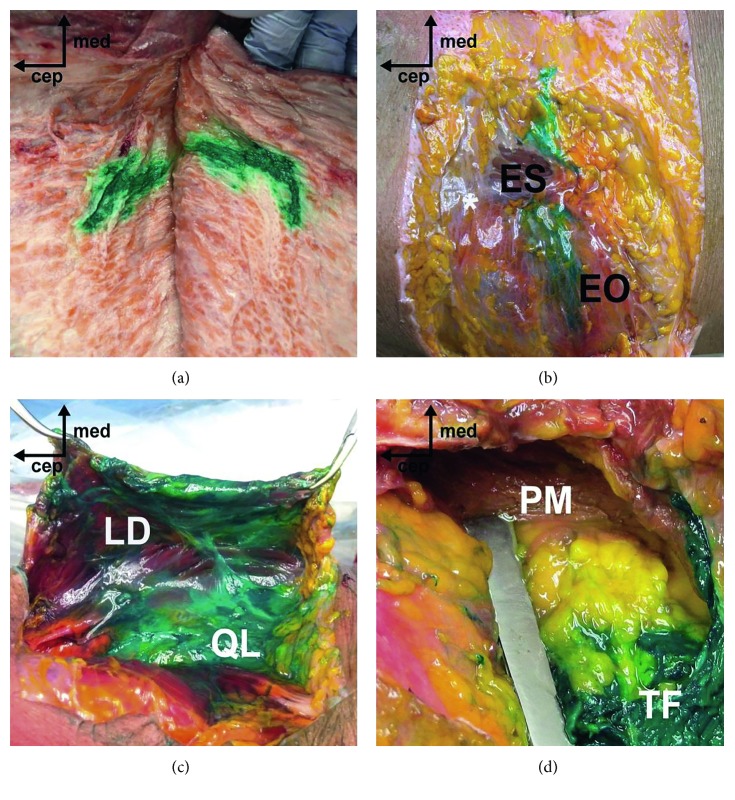
Cadaveric dissection following conventional lateral and posterior QL blocks. (a) Dye-stained subcutaneous tissue in a posterior QL block. (b) Intramuscular dye infiltration of the EO in a lateral QL block. (c) A successful, deeply stained middle layer of the TLF; however, the posterior layer of TLF below the LD was also stained in this posterior QL block. (d) The TF plane was stained, but the PM was not involved, in a lateral QL block. (ES, erector spinae; LD, latissimus dorsi; QL, quadratus lumborum; EO, external oblique muscle; PM, psoas major; TLF, thoracolumbar fascia; TF, transversalis fascia).

In total, 2 lateral QL blocks and 3 posterior QL blocks were performed (Figure 3). All lateral QL blocks (100%, 95% CI 0.197–1.000) and two of the three posterior QL blocks (66.6%, 95% CI 0.125–0.982) showed a certain extent of the lateral dye spread within the TAP, but one of the posterior QL blocks showed a predominantly medial dye spread. All of the lateral and posterior QL blocks (100%, 95% CI 0.462–1.000) demonstrated deep staining of the middle layer of the TLF, but slightly fainter deep staining of the anterior layer of TLF was observed. The fascia around the psoas muscle was not involved. One of the lateral QL blocks (50%, 95% CI 0.026–0.973) was associated with dye spread within the TF plane, and the other lateral QL blocks showed partial spread of the dye within the plane between the EO and IO and intramuscular staining of the EO. A small amount of dye was injected into the QL in all lateral blocks. Two of the three posterior QL blocks (66.6%, 95% CI 0.125–0.982) demonstrated spread of the dye along the posterior layer of TLF, and one of these blocks was clearly associated with subcutaneous tissue infiltration by the dye. The cephalad-to-caudal spread pattern was similar between the lateral and posterior QL blocks. There were no cases which involved dye spread above the level of the 12th rib or below the iliac crest level.

**Figure 4:**
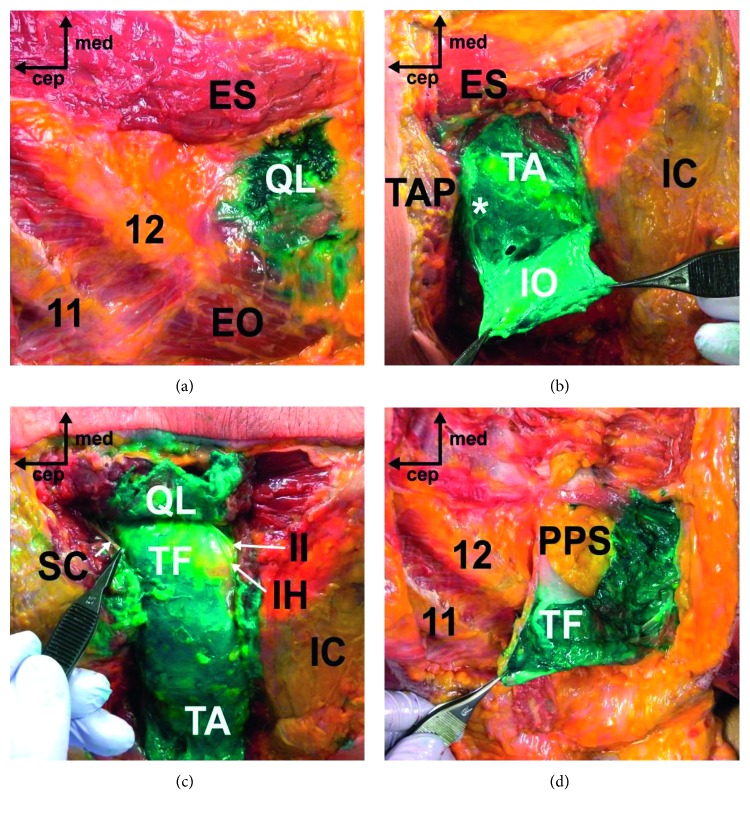
Cadaveric dissection following the posterior QL block with an alternative needle approach. (a) The deeply stained middle layer of the TLF. (b) The dye spread laterally to the TAP. (c) The subcostal (SC), iliohypogastric (IH), and ilioinguinal (II) nerves were stained. (d) The TF plane was deeply stained, but the PPS was preserved (ES, erector spinae; QL, quadratus lumborum; IC, iliac crest; EO, external oblique muscle; IO, internal oblique muscle; TA, transversus abdominis muscle; PPS, posterior pararenal space; ^*∗*^TAP, transversus abdominis plane; TLF, thoracolumbar fascia; TF, transversalis fascia).

In total, 5 posterior QL blocks with posteroanterior needle trajectory (an alternative approach) were performed (Figure 4). All this alternative QL blocks (100%, 95% CI 0.462–1.000) were associated with a certain amount of staining of the TAP, and two of them showed a deeply stained TAP. The spread of dye to the anterior and middle layers of the TLF was observed in all blocks (100%, 95% CI 0.462–1.000), but the fascia around the psoas muscle was not involved. Three of them demonstrated deep staining of the TF plane (60%, 95% CI 0.170–0.927), but the posterior pararenal space was preserved. There were no cases that showed dye spread to the posterior layer of the TLF, the subcutaneous layer, or intramuscular injection. The cephalad-to-caudal spread pattern was overall similar to that seen in the conventional QL blocks. One block demonstrated deep staining of the 11th intercostal nerve, but there was no dye infiltration above the 10th thoracic level in all cases. One block that involved staining of the TF showed dye spread below the iliac crest towards the iliac fossa, but the femoral nerve was not involved.

The subcostal, iliohypogastric, and ilioinguinal nerves were mostly involved, and there was no statistically significant difference between conventional and alternative approaches (*U*=10.500, *P*=0.690). Additionally, the thoracic paravertebral space and lumbar plexus within the psoas muscle were not affected in all blocks regardless of approaches.

## 4. Discussion

This cadaveric study demonstrated that the posterior QL block with posteroanterior needle trajectory can achieve a comparable extent of dye spread to conventional QL blocks, although this alternative approach was more frequently associated with the spread to the TAP. The spread to the subcutaneous layer or intramuscular infiltration was very rarely observed in this alternative approach. Regardless of the needle trajectory used, the thoracic paravertebral space and lumbar plexus were not stained.

In this study, there was not much variation in the medial spread of dye surrounding the QL between different approaches for the QL block, although our alternative approach showed the spread of dye within the TAP with greater frequency. Deep staining of the middle layer of the TLF was observed across most blocks, regardless of the needle approach used. However, the anterior layer of the TLF, especially in the fascia between the QL and psoas muscle, showed relatively insufficient staining in both approaches. Nonetheless, the alternative QL blocks were more frequently associated with the spread of dye from areas anterior to the QL to those lateral to the flank along the TF plane.

Overall, we observed the spread of dye to the anterior and middle layers of the TLF in conventional QL blocks. However, despite accurate needle tip placement, unintended dye spread along the posterior layer of the TLF or the LD surface towards the subcutaneous layer was observed, especially in conventional posterior QL blocks. Also, some intramuscular dye infiltration into the abdominal wall muscles or the QL was also observed in lateral QL blocks. Indeed, these spread patterns of dye were similarly observed in a previous cadaver study [7]. In lateral QL blocks, there is a possibility of piercing the lateral margin of the QL or abdominal wall muscles with the needle. The spread of dye back to the TAP and subsequent leaking to abdominal wall muscles are other possible scenarios. In conventional posterior QL blocks, despite accurate needle placement at the LIFT, the injectate spread could preferentially occur to the posterior vertex of the LIFT and then leak posteriorly to the subcutaneous layer along the LD. As shown in our results, spread to the subcutaneous layer or intramuscular infiltration was seldom observed in posterior QL blocks with a different needle trajectory. Because the needle tip is advanced towards the lateral vertex of the LIFT over the QL on the sonographic image when performing a posterior QL block with posteroanterior needle trajectory, the chance of spread to the subcutaneous layer or intramuscular infiltration seems to be low.

The LIFT, the boundaries of an adipose-filled region formed by the paraspinal retinacular sheath and the anterior and posterior laminae of the TA aponeurosis, may be a potential conduit for spread of injectate from the lumbar to the thoracic paravertebral space [8]. Also, histologically, the TLF contains a high density of nociceptive fibers [9]. This anatomical characteristic was sometimes used as an explanation for the superiority of posterior QL blocks over lateral QL or TAP blocks [10]. Although we hypothesized that dye injection in the LIFT would spread cranially to the thoracic paravertebral space, our results showed the contrary. A very recent cadaveric study also reported limited thoracic spread following posterior QL blocks similar to our study [3]. Considering the cadavers used in this study, which lack diaphragm or any other muscle movements, the spread of injectate may be more dynamic and extensive in living subjects. However, the estimated injectate volume that infiltrates into the thoracic paravertebral space appears to be too small for posterior QL blocks in living subjects, as well as cadavers [10]. In cases of anterior QL blocks, recent studies also have shown conflicting results regarding the thoracic spread [4, 6, 7]. Taken together, evidence supporting a central analgesic effect with visceral coverage during QL blocks still seems to be lacking [11].

In this study, there were no definite differences in nerve involvement patterns between QL blocks with different approaches. Most of the blocks, regardless of needle trajectory, were consistently associated with dye staining of the upper branches of the lumbar plexus, including the subcostal, iliohypogastric, and ilioinguinal nerves. The lumbar plexus within the psoas muscle was not involved. In living subjects, the extent of the cutaneous sensory block after a lateral QL block seems to be quite variable, with the sensory block only consistently achieved in the flank, groin, and upper lateral thigh [12]. Also, the assessment of the clinical effectiveness of lateral and posterior QL blocks seems to be more focused on their analgesic effects for surgery in T12-L1 dermatomes, such as low abdominal surgery, although there have been some case reports showing the analgesic effect of QL blocks for hip surgery [2, 10, 13, 14]. This study supported that lateral and posterior QL blocks, regardless of the needle approach used, might not always be a suitable replacement for lumbar plexus blocks. Also, our results confirmed that the block characteristics of lateral and posterior QL blocks might be quite different from anterior QL blocks [7, 11]. However, further studies using a caudally-oriented needle direction or a different injectate volume may be needed.

Regarding the technical aspect of these blocks, the optimal injection point seems to be more easily achieved when using the posterior QL block with posteroanterior needle trajectory when compared with the conventional method, in our experience. After, the overall posterior abdominal wall structure, including deeper structures such as psoas muscle and peritoneum, was elucidated using a convex probe (Figure 2(a)), the LIFT was readily identified using a linear probe because it is relatively superficially located (Figure 2(b)). Our alternative approach could allow for reaching the LIFT with a shorter needle path, whereas the conventional approach requires a longer needle path because the needle passes through the layers of the abdominal wall muscles in most cases. In conventional lateral QL blocks, the target injection point can sometimes be ambiguous in real clinical practice, because there are many anatomical structures, such as the anterior and middle layers of the TLF, multilayer of TA aponeurosis, and adjacent muscles, gathered around the anterolateral margin of the QL.

This study has some limitations. This study used a small sample size. Considering the cadaveric nature of this study, changes in tissue integrity could affect diffusion of the injectate. Also, in living subjects, the chest and abdomen movement during inspiration and expiration can lead to delayed injectate diffusion. In addition, factors such as temperature may affect diffusion, and we were unable to control for this.

## 5. Conclusion

In conclusion, the alternative approach in this study, a posterior QL block with posteroanterior needle trajectory, could achieve a comparable extent of dye spread, when compared to the conventional approach in soft-embalmed cadavers.

## Figures and Tables

**Table 1:** Comparison of dye staining between the different needle approaches for QL blocks.

Structure	Conventional lateral QL block (*n*=2)			Conventional posterior QL block (*n*=3)			Alternative posterior QL block (*n*=5)		
	Deep staining	Faint staining	No staining	Deep staining	Faint staining	No staining	Deep staining	Faint staining	No staining
*Thoracolumbar fascia*									
Anterior layer	1	1	0	1	1	1	2	2	1
Middle layer	2	0	0	3	0	0	5	0	0
Posterior layer	1	1	0	2	0	1	0	1	4

*Fascial plane*									
EO-IO plane	1	0	1	0	1	2	0	1	4
IO-TA plane (TAP)	1	1	0	0	2	1	2	3	0
TF plane	1	0	1	0	0	3	3	1	1

*Nerves*									
T11 root	0	0	2	0	0	3	1	0	4
Subcostal (T12)	2	0	0	1	1	1	4	1	0
Iliohypogastric	2	0	0	2	1	0	4	1	0
Ilioinguinal	2	0	0	2	1	0	4	1	0
Genitofemoral	0	0	2	0	0	3	0	0	5
Lateral femoral cutaneous	0	0	2	0	0	3	1	0	4
Lumbar plexus	0	0	2	0	0	3	0	0	5

*Final extent of dye spread*									
Above the arcuate ligament	0	0	2	0	0	3	0	0	5
Below the iliac crest	0	0	2	0	0	3	1	0	4
Anterior to the psoas muscle	0	0	2	0	0	3	0	0	5
Subcutaneous layer	0	1	1	1	1	1	0	0	5

EO, external oblique muscle; IO, internal oblique muscle; TA, transverse abdominis muscle; TAP, transversus abdominis plane; TF, transversalis fascia.

## Data Availability

The data used to support the findings of this study are available from the corresponding author upon request.
